# Isolation of blood-derived extracellular vesicles for disease diagnosis: Methods, challenges, and applications

**DOI:** 10.1016/j.jpha.2026.101640

**Published:** 2026-04-17

**Authors:** Xi Song, Jacques Crommen, Marianne Fillet

**Affiliations:** Laboratory for the Analysis of Medicines (LAM), Department of Pharmacy, CIRM, University of Liege, Liège, 4000, Belgium

**Keywords:** Extracellular vesicles, Blood, Proteomics, Biomarkers

## Abstract

Extracellular vesicles (EVs) are a family of cell-derived membrane vesicles found in almost all body fluids. Blood-derived EVs are particularly attractive for clinical diagnostics due to their accessibility and systemic representation. However, the intrinsic complexity of EVs and the abundance of non-EV particles in blood have hindered the establishment of a standardized isolation method. Meanwhile, research on EV isolation is rapidly evolving, and there is a clear need for reviews that synthesize the recent advances in this field, particularly those focusing on blood-derived EVs. In this review, we provide a comprehensive overview of both established and emerging isolation techniques, summarizing their advantages, limitations, and applications reported over the past years, to provide methodological insights and guidance for future isolation studies.

## Highlights

• Overview of EV isolation methods from blood samples.
• Comparison of established and emerging EV techniques.
• Pros and cons of current EV isolation strategies.
• Guidance for future EV method development and standardization.

## Graphical abstract

**Figure:**
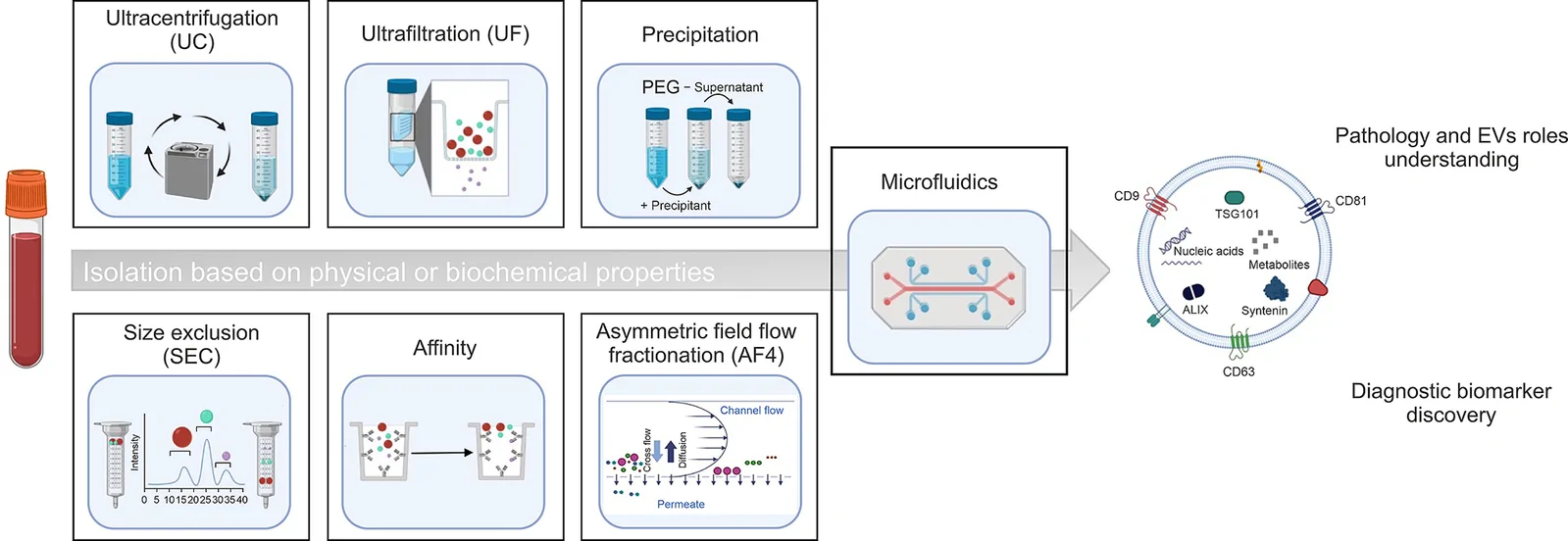

## 1 Introduction

Extracellular vesicles (EVs) are membrane-bound subcellular particles actively secreted by cells. They are ubiquitously present in various biological fluids, including blood, urine, and saliva [1]. Based on their biogenesis and physical properties, EVs are commonly categorized into exosomes (30–150 nm), microvesicles (100–1000 nm), and apoptotic bodies (500–5000 nm) [2,3]. However, due to a substantial overlap in terms of size, density, and composition (Fig. 1), and the current lack of reliable markers to distinguish between subtypes, the International Society for Extracellular Vesicles (ISEV) recommends the use of the generic term “EVs” unless the vesicle's origin is clearly defined [4]. This standardized nomenclature provides a unified foundation for subsequent methodological evaluation and functional investigations.

**Fig. 1:**
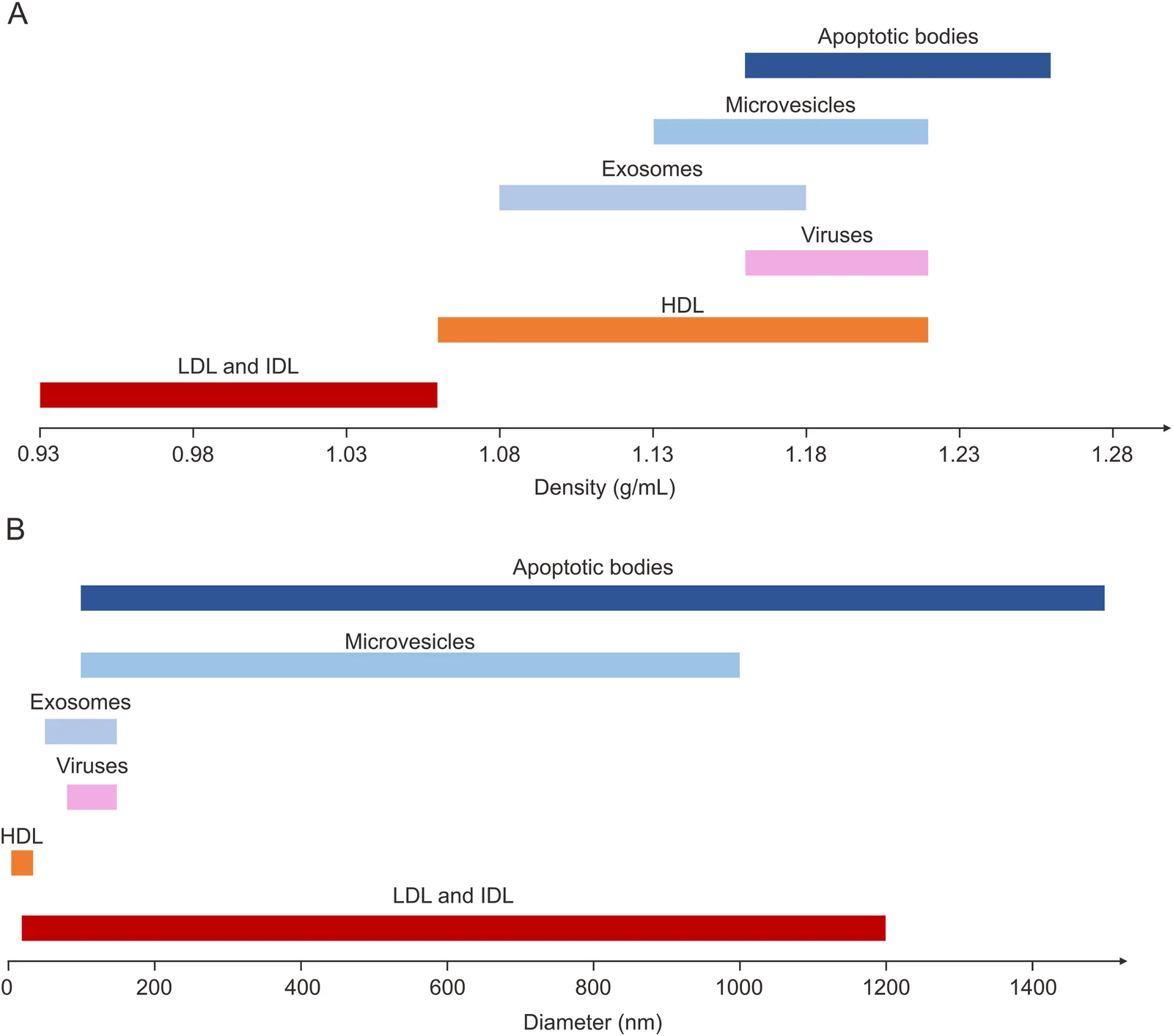
Overview of the density and diameter distributions of extracellular vesicles (EVs) and non-EV blood particles. (A) Density distribution of EVs and non-EV blood particles. (B) Diameter distribution of EVs and non-EV blood particles. HDL: high-density lipoprotein; LDL: low-density lipoprotein; IDL: intermediate-density lipoprotein.

Once considered cellular waste [5,6], EVs are now recognized as key players in a variety of physiological and pathological processes. By carrying proteins, lipids, RNAs, and DNAs, EVs mediate intercellular communication and participate in immune regulation, tissue repair, tumor progression, and metabolic reprogramming [1]. Their membrane stability, traceable cellular origin, and ability to reflect the state of the secreting cells make EVs attractive biomarker carriers. Compared to free-circulating proteins or nucleic acids, the encapsulated molecular contents of EVs offer greater stability and functional integrity in biofluids, supporting their potential for liquid biopsy and early disease detection [7,8].

Among all biofluids, blood is particularly advantageous for clinical applications because it is minimally invasive, easy to collect, and reflects systemic physiological and pathological conditions [9]. As a key liquid biopsy medium, blood contains a diverse pool of EVs carrying molecular information from multiple tissues. These plasma-derived EVs are gaining increasing attention as promising tools for disease diagnosis, prognosis monitoring, and therapeutic response assessment [10].

Recent studies have demonstrated the diagnostic potential of plasma EVs in diverse disease contexts. In cancer, EVs are enriched with tumor-derived membrane proteins, such as cluster of differentiation 147 (CD147) and epithelial cell adhesion molecule, which show high diagnostic sensitivity in breast and pancreatic cancers [11,12]. In neurological disorders, neuron-specific EV proteins such as L1 cell adhesion molecule and excitatory amino acid transporter 2 (EAAT2) have been applied in the monitoring of Alzheimer's disease and multiple sclerosis [13,14]. In autoimmune and inflammatory diseases, plasma EVs carrying proteins like S100 calcium-binding protein A9 and serum amyloid A1 are significantly elevated in newly diagnosed rheumatoid arthritis, supporting their use in disease stratification [15]. Compared with free proteins, EV-encapsulated proteins exhibit higher cell-type specificity and greater stability in circulation, reinforcing their value in the context of clinical proteomic analysis [16].

However, the effective detection of EV proteomes heavily relies on high-purity and high-yield EV isolation during sample preparation. Compared with low-background samples like cell culture media or urine, plasma is a highly complex matrix enriched with lipoproteins, soluble proteins, and cellular debris. These contaminants frequently co-isolate with EVs and interfere with downstream mass spectrometry analysis, reducing analytical sensitivity and specificity [17,18]. Furthermore, disease-relevant EV subpopulations are typically present in low abundance, requiring selective and efficient isolation approaches [19]. Thus, the development of robust, reproducible, and proteomics-compatible isolation methods tailored to plasma remains essential for clinical translation.

Although several reviews have summarized EV isolation strategies, most of them have focused on general workflows and physical principles, with limited evaluation of plasma-based methods in the context of proteomics and disease diagnostics [20]. As the field continues to advance rapidly, there is a growing need for an updated synthesis of current approaches. Unlike previous reviews, this work focuses specifically on reports dealing with blood-derived EVs published over the past years, with an emphasis on proteomics-oriented studies, clinical relevance, and methodological trade-offs. By analysing representative studies, we aim to provide practical insights and guidance to support the development of reproducible and clinically compatible EV isolation workflows.

## 2 Complexity of blood-derived EVs and non-EV contaminants

As one of the most used sample types in clinical liquid biopsy [21], blood is an inherently complex biofluid and can be more accurately regarded as a liquid form of tissue matrix. Due to the high abundance of cellular components in whole blood (accounting for approximately 50% of the circulating volume) [22], it is almost impossible to study EVs directly from whole blood. Consequently, current research on blood-derived EVs typically relies on plasma or serum as the starting material. The complexity of blood poses substantial challenges for EV isolation and downstream analyses, and this complexity arises mainly from three interrelated factors. First, EVs exhibit high intrinsic heterogeneity. In blood, EVs are secreted by a broad range of cell types, including platelets, erythrocytes, leukocytes, endothelial cells, as well as non-hematopoietic tissues such as liver, muscle, and adipose tissue [23,24]. These diverse origins result in substantial variation in vesicle size, composition, and molecular cargo. Importantly, functionally specific EVs, such as tumor-derived vesicles, often constitute only a minor fraction of the total EV population, making their enrichment and identification particularly challenging [25]. Second, the release and composition of EVs are highly dependent on physiological and pathological conditions. Factors such as inflammation, infection, malignancy, or intense physical activity can markedly alter EV secretion and molecular profiles [26,27]. Even within a single individual, EV populations may differ substantially across time points, introducing a considerable variability that complicates the reproducibility of isolation protocols [18]. Third, preanalytical procedures can further exacerbate this variability. Common factors such as the type of anticoagulant, storage temperature and duration, freeze/thaw cycles, and centrifugation settings during plasma or serum preparation may inadvertently trigger platelet activation or compromise EV integrity, thereby affecting the reliability of downstream analysis [28,29].

Beyond the inherent heterogeneity and dynamic responsiveness of EVs, blood samples are also rich in non-EV particles that frequently co-isolate with EVs and interfere with subsequent omics-based analyses or biomarker discovery efforts. These interfering components can be broadly categorized into three groups: platelet activation–induced particles (e.g., platelet-derived EVs enriched in glycoprotein IIb/IIIa (GPIIb/IIIa), glycoprotein Ib–IX–V complex (GPIb-IX-V), and α-granule proteins), abundant coexisting macromolecules (e.g., albumin, fibrinogen, immunoglobulins), and potential exogenous vesicular contaminants (e.g., virus-associated vesicles).

Studies have shown that approximately 30% of blood EVs originate from erythrocytes and about 20% from leukocytes, whereas platelets, being the most prevalent anucleate cells in circulation, can contribute up to 50%–90% of the large EV fraction in healthy individuals. Elevated platelet counts are often associated with cardiovascular diseases and certain cancers, suggesting that platelet-derived EVs may carry clinically relevant signals under specific pathological conditions. However, platelets are highly sensitive to mechanical and chemical stimuli; during blood collection, mixing, centrifugation, and other pre-analytical procedures, they are easily activated and release substantial amounts of EVs *ex vivo*. These vesicles do not reflect the *in vivo* disease process but largely represent pre-analytical, procedure-induced artifacts [[30], [31], [32]].

Zhang et al. [33] provided experimental evidence for this phenomenon by systematically characterizing the profiles of blood-derived EVs from mouse plasma and serum using nanoparticle tracking analysis (NTA) and proteomic analysis. Their results revealed that EV fractions isolated from serum contain additional EVs released from platelets during coagulation reactions occurring during serum preparation, leading to compositional differences compared to plasma-derived EVs. Further proteomic analysis showed that EV fractions from serum were highly enriched in platelet-associated proteins, including platelet-specific receptor complexes such as GPIIb/IIIa and GPIb-IX-V, α-granule proteins, and other proteins dominantly expressed in platelets.

Although various centrifugation strategies are commonly used to reduce residual platelets in plasma and serum, complete removal remains difficult to achieve, and the extent of platelet depletion is often insufficiently reported in many studies. Incorporating an additional filtration step after centrifugation can further decrease the number of remaining platelets, thereby reducing contamination from platelet-derived EVs that are released *ex vivo* during sample handling [34].

In parallel, abundant plasma proteins such as albumin, immunoglobulins, and fibrinogen share overlapping physical properties with EVs and are prone to co-enrichment during ultracentrifugation (UC), filtration, or chromatographic steps. These proteins may also adsorb onto EV surfaces, forming a “protein corona” [35,36] that alters vesicle behavior and confounds quantitative analysis. In addition, plasma contains protein and ribonucleoprotein aggregates that can likewise enter EV fractions and interfere with subsequent analyses.

Lipoproteins are spherical particles responsible for transporting major lipids in the bloodstream. They consist of an amphipathic surface layer composed of proteins, free cholesterol, and phospholipids, which encloses a hydrophobic core containing cholesteryl esters and triacylglycerols [17]. Based on their lipid and protein composition, circulating lipoproteins can be classified into high-density lipoproteins (HDL), low-density lipoproteins (LDL), intermediate-density lipoproteins (IDL), very-LDL, lipoprotein(a), chylomicrons, and chylomicron remnants [37].

These lipoproteins exhibit substantial overlap with EVs in terms of size, density, and molecular composition (Fig. 1). Moreover, many lipoprotein subclasses circulate at levels several orders of magnitude higher than EVs, making them highly prone to co-isolation during standard procedures such as UC, density-gradient centrifugation, or size-exclusion chromatography (SEC). As a result, lipoproteins are among the most challenging contaminants to remove from EV preparations, and this issue becomes more pronounced in individuals with metabolic disorders, cardiovascular disease, or inflammatory conditions, where lipoprotein levels are typically elevated. Notably, their concentrations vary considerably between individuals and fluctuate within the same individual depending on physiological factors such as feeding status, adding further complexity to EV analysis [18,38].

Therefore, studies requiring higher-purity EV preparations often rely on combining multiple separation approaches based on distinct physical or biochemical principles, such as SEC, density-gradient centrifugation, or affinity-based depletion, to more effectively minimize lipoprotein interference [39]. To assess the extent of lipoprotein contamination, levels of specific apolipoproteins, such as apolipoprotein AI (ApoA1) for HDL and apolipoprotein B (ApoB) or apolipoprotein E for other lipoprotein classes, can be measured by enzyme-linked immunosorbent assay (ELISA) or Western blot, providing useful indicators of the purity of EV preparations.

Additionally, in pathological or non-sterile conditions, co-isolation of viral particles, virus-associated EVs, and even vesicles of bacterial or fungal origin may occur. Viruses often share similar membrane structure, size range, and biophysical characteristics with EVs, making their separation particularly challenging. Notably, many enveloped viruses can hijack the host EV biogenesis pathways, particularly the endosomal sorting complexes required for transport machinery, to facilitate their dissemination, thereby increasing the background complexity and reducing the sample purity [40]. Some studies have reported the presence of viral components such as hepatitis C virus glycoproteins and Ebola virus nucleoproteins within isolated EVs [41,42]. In addition, increased levels of the host protein STING (stimulator of interferon genes) have been observed in CD9^+^ EVs following herpes simplex virus 1 infection [43]. These virus-associated EVs carrying heterogeneous viral or host components may resemble endogenous EVs, potentially confounding downstream omics analyses and biomarker discovery.

These aspects are illustrated in Fig. 1, which depict the density and diameter distributions of EVs and non-EV contaminants in blood. Therefore, achieving high-purity EV isolation from blood requires a detailed understanding of these interfering components and the strategic selection or combination of isolation techniques tailored to specific research objectives, to achieve relatively higher EV purity and yield for downstream proteomic analysis.

## 3 Traditional approaches for EV isolation

In this paper, we have reviewed the most employed conventional methods for isolating EVs from plasma, including UC, SEC, ultrafiltration (UF), polymer-based precipitation, and immunoaffinity capture. These techniques have been widely applied in EV research and clinical sample processing, each offering distinct advantages and limitations.

Table 1 provides a comparative summary of these traditional methods based on key analytical performance criteria, such as EV purity, yield, specificity, time efficiency, scalability, proteomics compatibility, and accessibility. This overview offers a practical reference for selecting appropriate workflows tailored to specific sample types and research goals.

**Table 1:** Comparative assessment of conventional extracellular vesicle (EV) isolation methods for plasma-derived samples based on key analytical performance criteria.

Isolation method	Purity	Yield	Specificity	Time efficiency	Scalability	Proteomics compatibility	Accessibility
Ultracentrifugation	b	c	a	a	c	b	c
Size-exclusion chromatography	c	b	a	c	b	c	b
Ultrafiltration	a	b	a	c	b	a	c
Polyethylene glycol	a	c	a	c	c	a	c
Immunoaffinity capture	c	a	c	b	a	c	a
Asymmetrical-flow field-flow fractionation	c	b	b	a	a	c	a

a Low.

b Moderate.

c High.

### 3.1 UC

UC is the most widely used technique for isolating EVs [44]. This method separates particles based on differences in size and density through a series of centrifugation steps. Low-speed centrifugation is first applied to remove cells, debris, and large vesicles, followed by high-speed UC to pellet EVs. The resulting pellet is typically washed with phosphate-buffered saline and re-centrifuged to minimize protein contamination [44,45]. Key centrifugation parameters, such as force, duration, and rotor type, are adjusted based on the specific experimental goals [46]. In UC workflows, the choice of rotor type is critical. Swinging-bucket and fixed-angle rotors differ in sedimentation behavior, and rotor type together with g-force and run time significantly affects EV yield and purity; longer runs can increase yield but overly long runs increase protein carryover [47,48]. The entire process is usually conducted at 4 °C to maintain EV structural integrity and reduce enzymatic degradation.

UC remains the benchmark method for isolating EVs from plasma, particularly in proteomics-oriented studies. It can process relatively large sample volumes [49] (≥3 mL) and enables sample concentration by generating a pellet, making it suitable for downstream analyses requiring high protein input. In addition, UC is relatively low-cost, does not rely on commercial kits, and is adaptable to a wide range of biofluids, with a typical workflow requiring approximately 4 h [50] from sample loading to EV pellet collection. Due to its technical maturity and widespread accessibility, it is frequently used for performance evaluation and benchmarking of emerging isolation techniques [7].

Numerous studies have demonstrated that UC and its optimized variants offer higher EV purity and broader protein coverage than alternative methods (such as SEC, polyethylene glycol (PEG) precipitation and immunoaffinity capture). For example, Wan et al. [51] compared various EV isolation strategies and showed that optimized UC significantly reduced protein contamination compared to phosphatidylserine (PS)-based affinity capture and commercial kits (exoEasy Maxi Kit using membrane affinity; Exo-spin midi using size exclusion chromatography; ExoQuick and miRCURY kits using polymer precipitation). Compared to conventional two-step UC, the optimized UC protocol yielded higher EV purity, with markedly reduced plasma contaminants such as human serum albumin (HSA), immunoglobulin G (IgG), and ApoA1, and enabled deeper proteomic profiling, identifying on average 1025 proteins versus 706 by the conventional method in healthy donor plasma. It also enriched EV markers including CD9, Flotillin-1, HSP90, and SDCBP, as well as exosome signature proteins. Although several commercial kits showed higher recovery rates of CD9^+^ EVs, their relative purity was two to three orders of magnitude lower than that achieved by the optimized UC, highlighting the superior suitability of the latter for proteomic applications. This result was further validated in plasma samples from pancreatic cancer patients, where optimized UC yielded a significantly higher number of identified proteins, enriched EV markers (CD9, Flotillin-1, HSP90, SDCBP), and significantly reduced major plasma contaminants (HSA, IgG, ApoA1) compared to traditional UC. Beyond oncology, UC has also been widely applied in other disease areas. For example, in neurodegenerative disease research, UC has been used to isolate EVs from plasma for proteomic analyses aimed at identifying Alzheimer's disease-associated biomarkers [52]. In cardiovascular disease research, UC has been employed to isolate plasma EVs for lipidomic profiling, enabling the development of diagnostic models for acute myocardial infarction [53]. These studies highlight the importance of UC as a foundational method for biomarker discovery.

Despite its broad application, considerable inter-laboratory variation in UC parameters, such as centrifugal force, time, and rotor configuration, can compromise reproducibility. In a multicenter study involving four laboratories, Crigna et al. [54] compared EV isolation from conditioned medium of colorectal cancer cells using a standardized differential UC protocol, conducted in two independent rounds of isolation. The quantification and characterization of EVs were centrally performed by a single expert using Western blot, flow cytometry, NTA, and transmission electron microscopy, to minimize analytical variability. The study reported substantial differences in EV yield, protein content, and marker expression across laboratories, even under a standardized protocol, highlighting the impact of equipment and operator variability. The study workflow is shown in (Fig. 2A) [54], while Figs. 2B and C [54] illustrate representative NTA and Western blot results. The standardization of critical parameters, such as speed and k-factor, has therefore been strongly recommended. Moreover, UC may co-isolate abundant plasma proteins, such as lipoproteins and immunoglobulins, which can interfere with downstream omics analyses. Nonetheless, it continues to serve as the default reference method in comparative studies and remains fundamental in plasma-derived EV research.

**Fig. 2:**
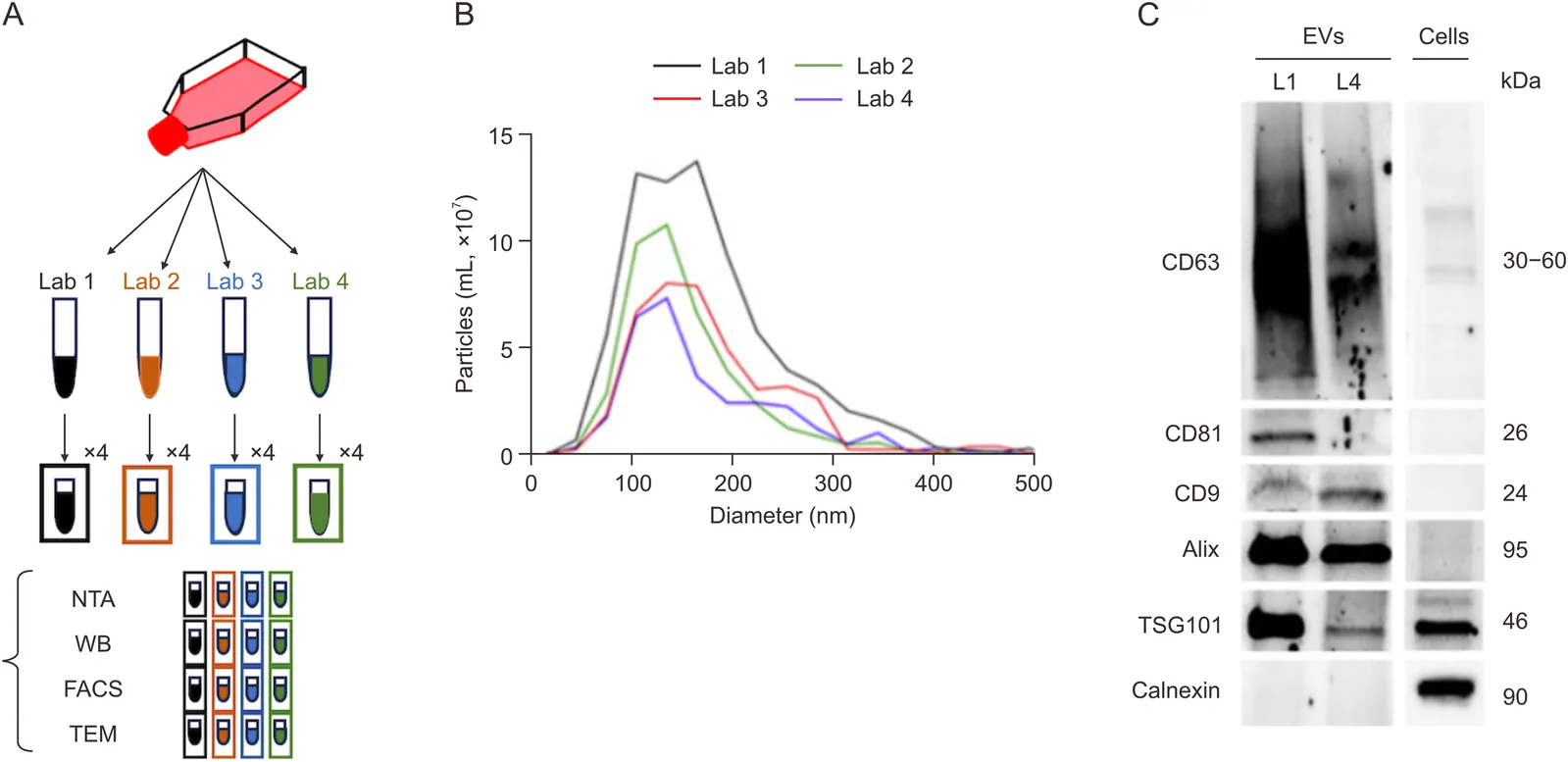
Overview of the workflow and inter-laboratory differences in ultracentrifugation (UC)-based extracellular vesicle (EV) isolation. Reprinted from Ref. [54] with permission. (A) Schematic workflow of EV isolation and characterization. (B) Size distribution of EVs obtained by four laboratories, showing differences in nanoparticle tracking analysis (NTA) profiles. (C) Western blot (WB) analysis of EV protein markers and the negative marker calnexin in EVs isolated by L1 and L4. L1–L4 denote independent preparations processed under identical conditions. FACS: fluorescence-activated cell sorting; TEM: transmission electron microscopy; CD63: cluster of differentiation 63; CD81: cluster of differentiation 81; CD9: cluster of differentiation 9; Alix: apoptosis-linked gene 2 (ALG-2)–interacting protein X; TSG101: tumor susceptibility gene 101.

### 3.2 SEC

In SEC, the biofluid sample is applied onto a porous gel filtration column and eluted with a physiological buffer as the mobile phase. Particles are separated based on their hydrodynamic size: larger particles, which cannot enter the pores, elute earlier via shorter paths, while smaller molecules penetrate more pores and elute later. The stationary phases typically consist of crosslinked polymers such as dextran (DEX), agarose, polyacrylamide, ethoxylated polyethylene oxide bonded to silica particles or cross-linked polystyrene-divinylbenzene, depending on the target size range and sample characteristics [[55], [56], [57]].

Since its first application to blood-derived samples by Böing et al. [55], SEC has rapidly become a mainstream technique for isolating EVs from plasma and serum. It is particularly well suited for EV-based biomarker discovery in clinical samples. The method enables a rapid separation of the sample components within 10–20 min without the need for specialized equipment, making it highly compatible with clinical workflows. In a comparative study, Askeland et al. [58] evaluated SEC, UC, and immunoaffinity capture, and reported that SEC achieved superior protein abundance and broader protein identification coverage in mass spectrometry analysis. Due to its minimal shear force, SEC also better preserves vesicle integrity and biological activity, offering distinct advantages over UC.

SEC has been broadly applied to various disease models. In the context of early diagnosis, Vanderboom et al. [59] developed a SEC-based EV isolation workflow to analyze plasma samples from patients with monoclonal gammopathy of undetermined significance and multiple myeloma. The study successfully identified protein biomarkers differentially enriched in small and large EV subpopulations, providing insight into EV-based stratification of plasma cell disorders. For disease monitoring, D'Ambrosio et al. [14] utilized SEC to achieve robust EV isolation from limited volumes of 6 mL plasma obtained from 11 relapsing remitting multiple sclerosis patients and 5 healthy controls, identifying EAAT2 as a potential marker of disease activity. These findings underscore the clinical applicability of SEC for high-throughput EV profiling in longitudinal studies.

Despite the many advantages of SEC in EV research, several limitations constrain its broader application. The relatively low resolution of the technique hampers its ability to separate specific EV subpopulations [60,61]. Moreover, SEC workflows remain insufficiently standardized in the field, with substantial procedural variability observed even within the same research group, thereby undermining data comparability and reproducibility [62]. In addition, certain sample types, such as cell culture supernatants and urine, often require pre-concentration prior to SEC to achieve adequate vesicle yields [63]. Depending on the intended downstream analyses, further reconcentration may be needed after purification. However, EVs may irreversibly adsorb onto membrane filters during these steps, resulting in significant loss of material. Addressing these technical challenges is essential to improve the reliability and scalability of SEC-based EV isolation strategies. Recent efforts have focused on optimizing column materials and pore structures and integrating SEC with real-time detection technologies such as ultraviolet–visible (UV–Vis) and turbidity sensors to improve separation efficiency and sample compatibility. For example, Kaddour et al. [64] proposed a particle purification liquid chromatography system that combines SEC with in-line monitoring, demonstrating its potential to efficiently separate EV subpopulations from complex biofluids.

### 3.3 UF

UF is a size-based separation method commonly employed for the enrichment of EVs from plasma. It is low-cost, operationally simple, and compatible with a wide range of sample volumes (approximately 0.2–200 mL, depending on the membrane format) [65,66]. When isolating EVs by microfiltration, membranes with pore sizes of 0.8, 0.45, 0.22, and 0.1 μm are typically used, which retain particles larger than approximately 800, 450, 220, and 100 nm, respectively (±20%) [67]. In clinical settings, UF is often used for rapid concentration of EVs to meet the input requirements for downstream proteomic or nucleic acid analyses.

Despite its practical advantages, UF suffers from two major limitations in plasma-based EV proteomics. First, abundant plasma proteins such as albumin and immunoglobulins, due to their comparable size to EVs, are co-retained during filtration, thereby increasing background noise and masking low-abundance EV-associated biomarkers. Second, the filtration membranes used in UF (e.g., polyethersulfone (PES)) exhibit nonspecific protein adsorption, which compromises EV recovery and quantification accuracy. Comparative studies have shown that regenerated cellulose membranes with a 10 kDa cutoff achieve the highest EV recovery, while PES membranes yield less than 40%, highlighting the influence of the membrane material on analytical outcomes [68].

As a result, UF is typically used as an auxiliary step rather than a standalone method. Its main applications include sample concentration, buffer exchange, and pre-treatment prior to other isolation methods such as SEC or immunoaffinity isolation. In recent years, tangential flow filtration (TFF) has been proposed as an improved UF approach, offering higher throughput and reduced membrane clogging [69]. In large-volume matrices such as cell culture supernatants, TFF has demonstrated enhanced reproducibility and yield [70]. However, in the context of limited volumes of plasma samples, the high dead volume and protein co-retention associated with TFF systems constrain their practical utility in plasma-based EV proteomics. In comparison, conventional centrifugal UF devices combined with SEC remain a more feasible and widely adopted strategy.

### 3.4 PEG

PEG precipitation involves adding a defined concentration of PEG (typically PEG6000 or PEG8000) to the sample, promoting EV aggregation through volume exclusion, followed by recovery via low-speed centrifugation. Generally, PEG with a molecular weight of 6000 to 20,000 Da is employed for EV precipitation. Low-molecular-weight PEGs (<5000 Da) are often used to promote the preferential hydration of proteins [71]. Originally developed for concentrating viruses and macromolecular complexes, this method has been widely adopted in EV research due to its simplicity and low cost. In plasma-based applications, PEG precipitation is often used as an initial enrichment step, particularly in settings with limited access to equipment or a need for simplified workflows [72,73]. Several commercial kits (e.g., ExoQuick) are based on this principle and are commonly employed for sample preparation prior to proteomic analysis.

However, the nonspecific nature of PEG often results in co-precipitation of abundant plasma proteins and lipoproteins, substantially interfering with downstream mass spectrometry sensitivity and proteome coverage [73]. As a result, PEG precipitation alone typically fails to meet the purity requirements for EV isolation and is frequently combined with other isolation methods such as SEC, density gradient centrifugation, or membrane filtration to improve the separation [74].

Notably, a recent study by Otani et al. [75] investigated the use of low-concentration PEG to reduce lipoprotein contamination. The authors demonstrated that 3% PEG could achieve satisfactory EV yield and sample purity while offering favorable cost and time efficiency compared to conventional methods. These findings suggest that under optimized conditions, low-concentration PEG may serve as an independent and viable strategy for plasma EV isolation.

A variant of PEG-based precipitation is the aqueous two-phase system (ATPS), typically formed by combining PEG with DEX. When the polymer concentration reaches a critical threshold, the mixture spontaneously separates into a PEG-rich top phase and a DEX-rich bottom phase, with proteins preferentially partitioning into the PEG phase and EVs being enriched in the DEX phase. ATPS features low interfacial tension, high water content, and excellent biocompatibility, and has been widely applied for cell and protein separation. Theoretically, this approach is capable of isolating all types of EVs and offers advantages such as low cost, operational simplicity, and scalability. However, its practical use in EV isolation remains limited, and concerns regarding purity, compatibility with downstream analyses, and potential interference from DEX residues still require further investigation [76,77].

### 3.5 Immunoaffinity capture

Immunoaffinity-based isolation relies on the specific binding between antibodies and surface antigens on the EV membrane. Antibodies are typically immobilized on solid supports such as magnetic beads, microplates, or microfluidic chips, enabling the selective enrichment of EV subpopulations expressing specific markers. This method is suitable for isolating EVs of defined origin or function and has been widely applied in biomarker discovery and disease-related studies [78,79].

Compared to physical separation techniques, immunoaffinity capture offers higher specificity and allows for the enrichment of low-abundance EVs from complex biofluids. However, this method has several limitations: its performance is highly dependent on antibody quality, and nonspecific adsorption or the formation of a protein corona can reduce selectivity. In addition, the recovery rate is generally low, and the captured EV subpopulation may not reflect the overall EV landscape, which limits its utility in large-scale studies [78,80].

Multiple studies have demonstrated that this method enables the enrichment of high-purity EVs from plasma, supporting high-throughput mass spectrometry and biomarker screening. For example, Suresh and Zhang [81] compared nine EV isolation methods and found that immunoaffinity capture yielded samples with narrower size distribution, higher purity, and broader proteomic coverage. This technique can also be combined with other methods, such as SEC or PEG, to enhance selectivity and reduce background interference. A comparison of these EV isolation methods and their proteomic outcomes is presented in (Fig. 3) [81]. Sharma et al. [82] applied SEC followed by Chondroitin sulfate proteoglycan 4 (CSPG4) antibody-based capture to isolate tumor-derived EVs from melanoma patient plasma, while Nazarova et al. [83] integrated a polymeric phase system with antibody enrichment to isolate colon epithelium-derived EVs for disease analysis. In recent years, a growing number of commercial kits and automated platforms have been developed to support the standardization and broader adoption of immunoaffinity-based EV isolation.

**Fig. 3:**
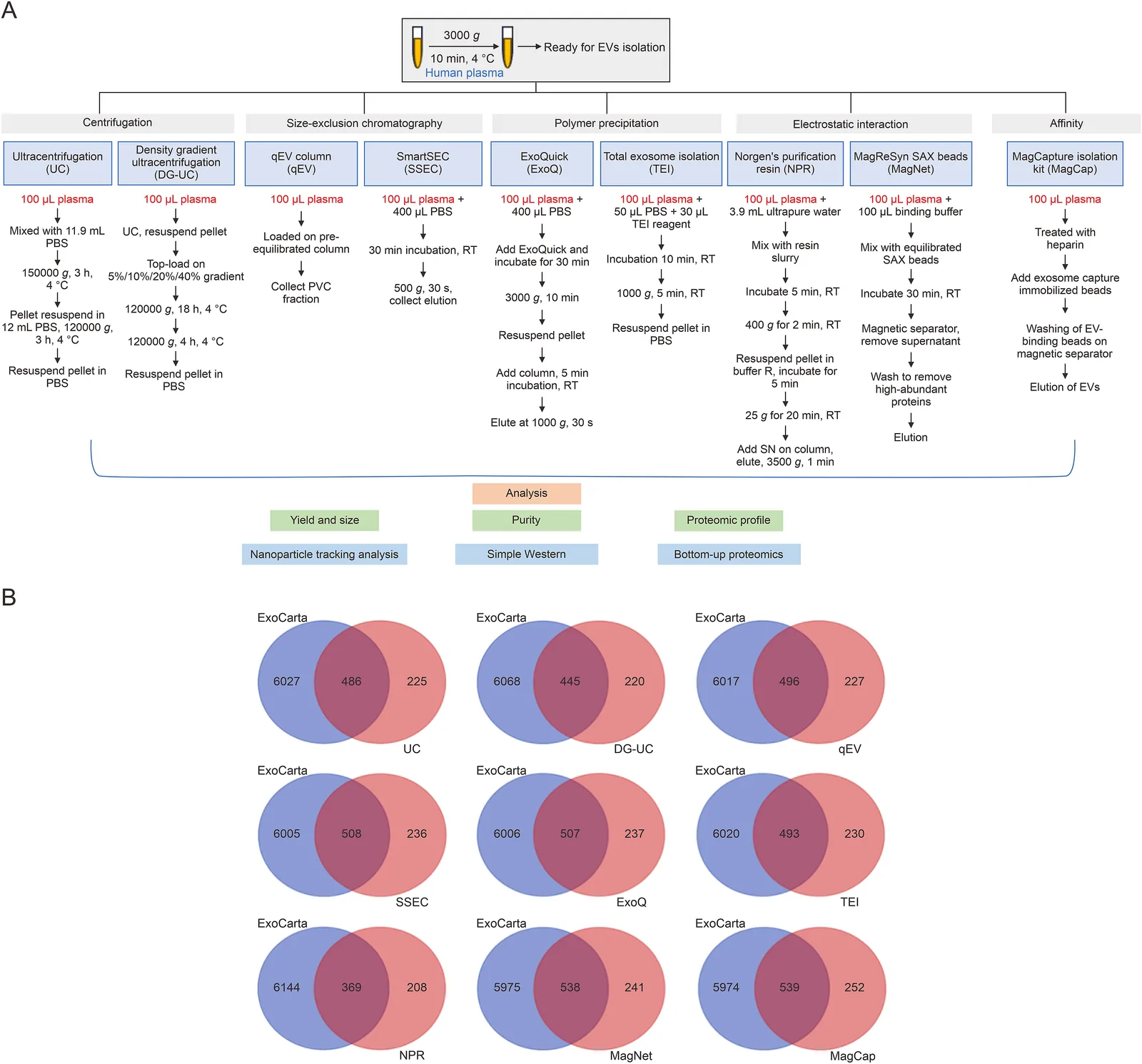
Comparison of extracellular vesicle (EV) isolation methods from human plasma. Reprinted from Ref. [81] with permission. (A) Schematic overview of the study design showing different EV isolation methods, including ultracentrifugation (UC), density gradient UC (DG-UC), size-exclusion chromatography (SEC), polymer precipitation, electrostatic interaction, and affinity. (B) Venn diagrams showing proteins identified in EV preparations by each method compared with the EV marker proteins listed in the ExoCarta database. SSEC: smart size-exclusion chromatography; PBS: phosphate-buffered saline; RT: room temperature.

Immunoaffinity capture is gradually evolving toward higher levels of standardization and automation. Future developments are expected to improve antibody affinity and the surface chemistry of capturing materials, thereby enhancing stability and performance. Despite challenges related to cost and generalizability, immunoaffinity capture continues to show strong potential in precision medicine [84].

## 4 Emerging technologies for EV isolation

While conventional EV isolation techniques remain widely used, challenges such as limited purity, low throughput, and poor reproducibility persist. To address these issues, considerable efforts have been made to develop alternative methods that offer improved separation, scalability, and sample integrity. Among these, asymmetrical-flow field-flow fractionation (AF4) and microfluidic technologies have gained increasing attention in recent years due to their unique advantages. The following sections provide an overview of these two emerging approaches.

### 4.1 AF4

AF4 is an open-channel technique that has been increasingly applied in EV isolation [85]. The separation principle of AF4 is illustrated in Fig. 4. It separates particles based on differences in diffusion coefficients using orthogonal laminar flows and avoids stationary phase packing, thereby minimizing shear stress and nonspecific adsorption. Tunable flow conditions enable high-resolution, size-dependent fractionation across a broad particle size range, while coupling with detectors such as UV or multi-angle light scattering (MALS) allows real-time, multiparametric analysis [86].

**Fig. 4:**
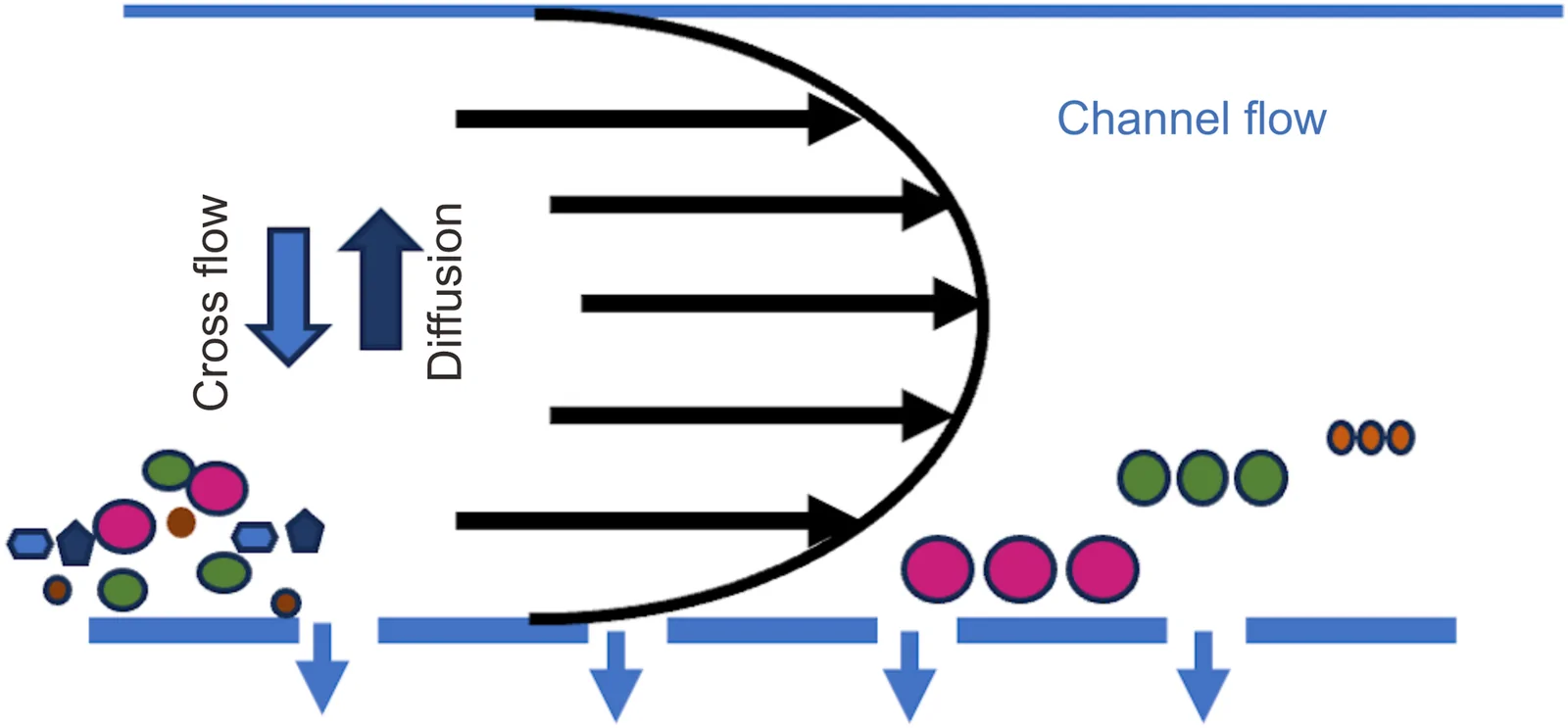
Schematic diagram of the asymmetrical-flow field-flow fractionation (AF4) separation principle.

Leveraging these advantages, AF4 is well suited for isolating EVs from complex biofluids such as plasma and serum. Wu et al. [87] optimized operational parameters to achieve high-purity EV isolation from human plasma, effectively removing HDL/LDL contaminants. This method was subsequently applied to EV proteomic studies in patients with β-thalassemia [88]. Zhang et al. [85] identified three EV subtypes, Exo-L, Exo-S, and exomeres in cell culture media using AF4. The same research group later extended this strategy to plasma samples by integrating AF4 with density cushion ultracentrifugation (DCU), enabling the separation of EV subpopulations for cancer biomarker discovery [89]. In 2023, the approach was further used to isolate tumor-derived EVs and investigate their role in liver metabolic dysfunction [90].

In addition, AF4 has been combined with other analytical techniques to improve separation performance. For example, Zhong's group coupled AF4 with capillary electrophoresis (CE) and large-volume sample stacking CE, improving EV purity and allowing for larger sample volumes, which significantly enhanced downstream quantification sensitivity [91,92]. Hu et al. [93] combined AF4 with DCU to isolate EVs from plasma and serum with minimal lipoprotein contamination, enabling the identification of numerous previously unreported EV-associated proteins. Holcar et al. [94] further demonstrated the feasibility of using AF4-UV-MALS for label-free quantification of EVs in human plasma, providing a standardized, non-destructive workflow with strong potential for clinical application.

Despite these advantages, AF4 still faces challenges, including sensitivity to sample conditions (e.g., pH, ionic strength, and the presence of stabilizing agents), dilution effects, limited resolution from LDL, and higher operational complexity compared to UC or SEC, requiring more expertise in method development and instrument handling. Future efforts should focus on optimizing resolution, improving buffer tolerance, and enhancing workflow automation, along with strengthening standardization to improve reproducibility and facilitate broader adoption [95,96].

### 4.2 Microfluidic platforms

Microfluidic technology manipulates fluids at the micrometer scale and offers advantages such as low sample consumption, rapid processing, and high integration with analytical workflows. In recent years, it has been widely applied to isolate EVs from plasma for proteomic analysis, becoming an important emerging approach that complements traditional methods. In essence, microfluidic devices do not establish entirely new separation principles; rather, they miniaturize and integrate existing approaches into a chip-based format, enabling multiple processes to be performed sequentially or in parallel. Common microfluidic EV isolation platforms fall into two main categories: surface marker-based immunoaffinity capture, which uses immobilized antibodies to specifically bind EVs carrying markers such as CD63, CD9, and CD81, and size-based filtration systems, which rely on microstructure designs to achieve separation according to particle size [97,98].

Studies have demonstrated the potential of microfluidics in EV separation and analysis. For example, Zhou et al. [99] developed a microfluidic chip for plasma separation and EV detection that integrates plasma pre-processing, EV enrichment, and protein detection in a single device, and successfully applied it to liquid biopsy of ovarian cancer patient samples. In another study, Nikoloff et al. [100] constructed a microfluidic system capable of profiling proteins in EVs secreted by single breast cancer cells. Notably, the EV protein expression profile from a single cell was found to be tissue-specific, effectively serving as a “tissue fingerprint” to aid disease identification.

Despite these advantages, microfluidic platforms for EV proteomics still face several challenges, including low throughput, insufficient standardization, and operational complexity, which limit their broader adoption. Future efforts should be focused on enhancing the versatility of these platforms and improving their compatibility with downstream analytical techniques, thereby promoting their standardized application in the analysis of clinical samples.

### 4.3 Lipid-targeted affinity-based isolation

EVs are made of a phospholipid bilayer enriched with diverse lipid species that not only maintain vesicle integrity but also carry characteristic information inherited from the plasma membrane of the parent cell. Variations in lipid composition and exposure patterns across different EV types provide potential molecular features for distinguishing specific EV subpopulations. Building on these properties, several strategies have been developed to exploit lipid–ligand interactions for EV enrichment, enabling affinity capture through selective recognition of membrane lipids as a complementary approach to traditional protein marker–based isolation. Current lipid-recognition ligands commonly include strategies based on lipid–lipid interactions, peptide–lipid binding, and other lipid-targeted affinity domains [101,102].

In recent years, increasing attention has been directed toward applying lipid-targeted ligands to directly capture EVs from plasma samples. For example, Zhang et al. [103] developed a 1,2-distearoyl-sn-glycero-3-phosphoethanolamine-functionalized magnetic bead platform capable of rapidly capturing plasma EVs within 10 min, demonstrating good reproducibility and broad proteomic coverage. This platform enabled high-throughput proteomic profiling of plasma-derived EVs and successfully identified several protein candidates associated with early-stage colorectal cancer, offering a fast, simplified, and cost-effective approach for EV-based clinical early detection.

In addition, Gori et al. [104] introduced a class of membrane-binding peptides designed to recognize specific lipid features on EV membranes. Integrated into a microarray chip platform, these peptides enabled direct capture and analysis of EVs from serum samples without the need for pre-isolation. Notably, these lipid-binding peptides exhibited superior binding performance compared with conventional anti-tetraspanin antibodies, highlighting their advantages of low cost and multifunctionality, and representing a promising direction for developing next-generation lipid-targeted affinity ligands.

Despite this progress, lipid-targeted affinity strategies still face several challenges in practical applications. The lipid composition of EVs is highly heterogeneous, and complex biofluids such as plasma contain abundant lipoproteins and non-EV lipid particles, making it difficult to balance specific binding with reduced background interference. Moreover, current lipid-targeting materials often have limited scalability and compatibility with automated systems, and their physicochemical properties may influence downstream mass spectrometry analysis or EV integrity. Future efforts may focus on improving ligand selectivity, integrating multifunctional materials, and advancing standardization and device automation to enhance isolation performance and support translation toward clinical diagnostics and single-particle analysis [105].

## 5 Current comparisons and performance assessments of blood-derived EV isolation approaches

In plasma-derived EV research, the choice of an isolation method directly affects the feasibility of downstream analyses and the quality of the results. Given the substantial differences between available techniques in terms of principle, yield, purity, and compatibility with downstream workflows, selecting the most appropriate strategy should be guided by the specific research objectives. For exploratory or early-phase studies, the primary goal is often to achieve high yield and throughput, enabling the recovery of as many EVs as possible from limited sample volumes for preliminary screening and hypothesis generation. In such cases, PEG-based precipitation and SEC are frequently used options. PEG precipitation generally provides the highest EV recovery among available methods but at the cost of lower purity, necessitating additional purification steps [106]. In contrast, SEC offers slightly lower recovery but can rapidly isolate EVs of higher purity from 1 mL of plasma while maintaining good compatibility with downstream analyses [107].

For studies aimed at biomarker discovery or requiring highly pure EVs, the emphasis shifts from maximizing yield to achieving specificity and purity. Immunoaffinity capture, for example using antibodies against CD9, CD63, or CD81, enables the selective enrichment of functionally relevant EV subpopulations while effectively removing plasma protein contaminants. This high specificity makes it particularly suitable for targeted analyses or validation of known biomarkers, although the overall EV yield is typically low [80,107]. Differential UC remains a widely used approach for processing larger sample volumes and producing moderately pure EV preparations. However, UC often co-pellets abundant plasma proteins and lipoproteins, which may compromise downstream analytical sensitivity, and its multi-step protocol is time-consuming [108]. Consequently, UC is frequently combined with additional purification steps to reduce contaminants while retaining adequate EV recovery.

In proteomic analyses of plasma-derived EVs, obtaining samples with both high purity and broad proteome coverage is critical for ensuring data quality and the reliability of biological discoveries. However, no universally accepted single isolation method can simultaneously achieve both of the goals, and multi-step workflows are therefore increasingly explored. These combined strategies often outperform single methods by improving EV purity and reducing co-isolated protein and lipoprotein contaminants (Fig. 5) [108]. The choice of methods and their sequence of combination should be aligned with the specific research objectives and balanced against the sample volume to optimize yield and purity.

**Fig. 5:**
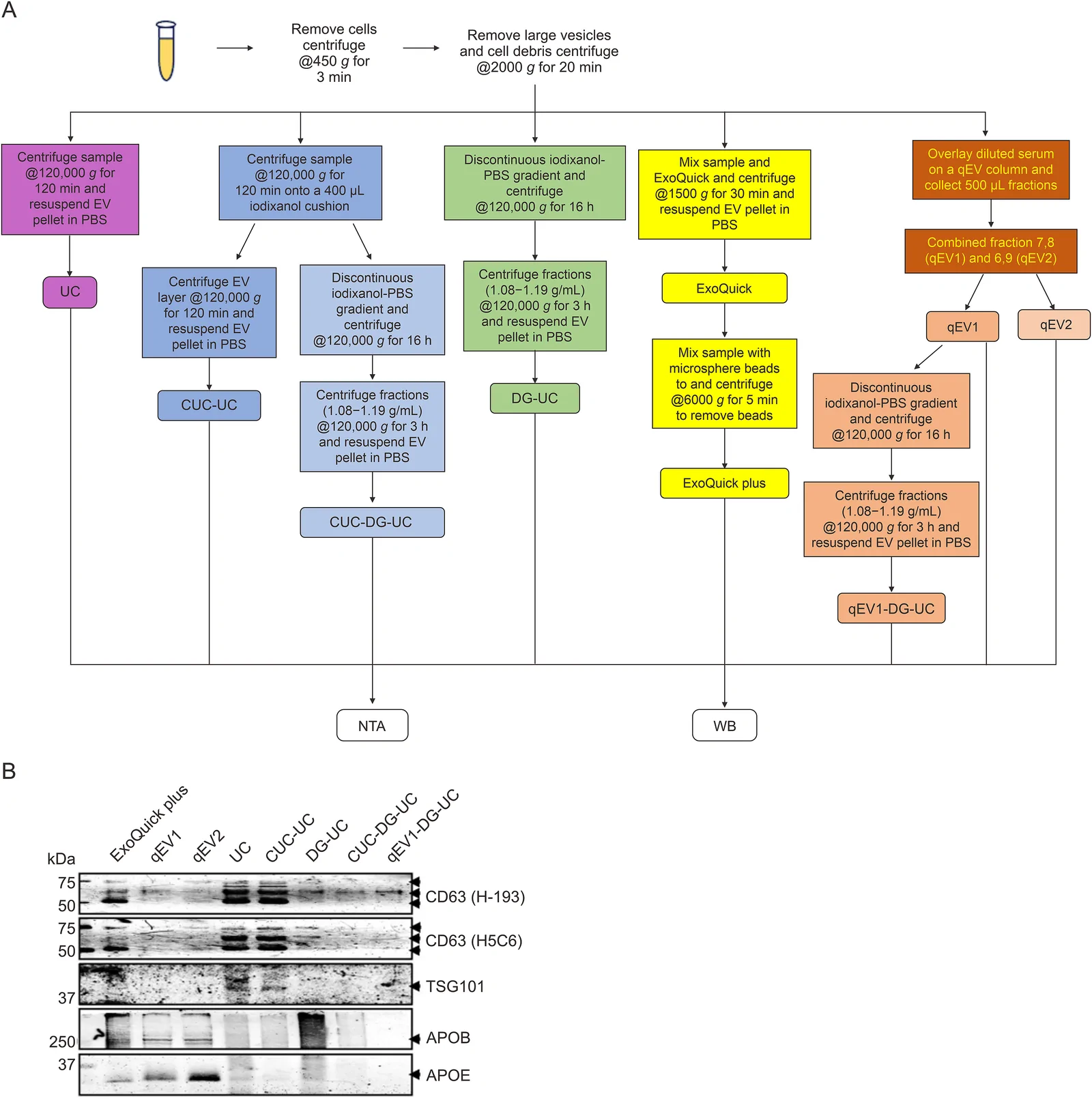
Comparison of extracellular vesicles (EVs) isolation methods from human serum. Reprinted from Ref. [108] with permission. (A) Schematic summary of EVs isolation from human serum using different methods alone or in combination. (B) Western blot analysis of EVs markers and lipoprotein markers. UC: ultracentrifugation; CUC: cushion ultracentrifugation; DG-UC: density gradient ultracentrifugation; CD63: cluster of differentiation 63; TSG101: tumor susceptibility gene 101; APOB: apolipoprotein B; APOE: apolipoprotein E. WB: Western blot; NTA: nanoparticle tracking analysis; PBS: phosphate-buffered saline.

For large-volume samples (typically >1–2 mL plasma), purity often takes precedence over yield, as sufficient EV quantities can be recovered from the available volumes. A common approach is to first apply SEC to remove most soluble high-abundance plasma proteins, thereby reducing sample viscosity, shortening subsequent centrifugation times, and minimizing co-pelleting of non-EV proteins during UC. In a proteomic study using 4 mL of plasma, Wei et al. [109] demonstrated that SEC followed by UC substantially reduced residual albumin and other plasma contaminants while increasing the detection of low-abundance EV proteins and enhancing proteome coverage.

For small-volume samples (e.g., <0.5 mL serum/plasma), the primary objective is often to secure sufficient EV quantities for downstream analysis, making yield a higher priority than purity. In such cases, an initial UC step can concentrate EV particles and remove some contaminants, followed by SEC for fine fractionation, thus retaining a balance of reasonable purity and high yield. Turner et al. [66] found that, for 200 μL serum, a UC + SEC workflow provided the best overall performance in terms of protein identification, purity, and particle recovery, outperforming SEC + UC and other combined methods.

Therefore, when designing multi-step isolation strategies, researchers should consider not only the overall research goals (e.g., exploratory analysis vs. biomarker validation) but also the sample volume, to determine the optimal method sequence and achieve the most appropriate trade-off between purity and yield.

In addition to selecting an appropriate isolation method based on the research objective and sample characteristics, it is equally essential to systematically evaluate the performance of the chosen approach in order to ensure the reliability of downstream analyses. Given the compositional complexity of plasma, the efficiency of EV isolation can be influenced by multiple factors, making it essential to assess the isolated products from multiple dimensions.

Among these evaluation parameters, purity is one of the most critical. High purity indicates that EVs are retained to the greatest extent while abundant plasma proteins (e.g., albumin, immunoglobulins) and lipoproteins, as well as other non-EV components, are effectively removed, thereby minimizing background interference in downstream analyses. The latest Minimal information for studies of extracellular vesicles (MISEV2023) [4] guidelines from the ISEV explicitly recommend assessing EV purity by detecting at least three well-recognized positive markers (e.g., CD9, CD63, CD81, tumor susceptibility gene 101 (TSG101), apoptosis-linked gene 2 (ALG-2)–interacting protein X (ALIX)) and at least one negative marker (e.g., albumin, ApoA1, ApoB, or other plasma protein and cellular debris markers), and by reporting the detection methods and signal intensities to ensure accuracy and comparability (MISEV2023). Such evaluations are typically performed using Western blot, ELISA, or flow cytometry, and are complemented by morphological analyses such as transmission electron microscopy (TEM) to confirm the specificity and cleanliness of the isolated products from multiple perspectives.

Yield refers to the number of EVs obtained from a given sample volume and is an important indicator of isolation performance. Common measurement approaches fall into two main categories: particle counting and protein quantification. Particle counting methods, such as NTA, typically estimate yield by comparing the total particle number before and after isolation; however, these techniques may overestimate results because they also detect non-EV particles such as lipoproteins and protein aggregates. In human plasma, the reported total concentration of EVs varies widely, ranging from 10^6^ to 10^13^ particles/mL, largely depending on the detection method: flow cytometry generally yields lower estimates, whereas NTA often reports higher values. Protein-based approaches (e.g., bicinchoninic acid (BCA) or Bradford assays) are commonly used in proteomics-focused studies but cannot distinguish between EV proteins and co-isolated plasma proteins, which may lead to overestimation in protein-rich biofluids. These values are strongly influenced by the isolation method, quantification technique, and sample-specific factors. The MISEV2018 guidelines recommend combining particle counts with the detection of EV-specific markers (e.g., CD9, CD63, TSG101) and negative markers (e.g., albumin, ApoA1) to provide a more accurate assessment of both yield and purity [22,110,111].

In addition to yield and purity, particle size distribution analysis is also critical for assessing whether the isolation method enriches the target EV subpopulation and whether particle aggregation or disruption has occurred. Common analytical techniques include NTA and TEM, which can be used to verify deviations from the intended size range. Furthermore, the compatibility of reagents used in sample preparation with mass spectrometry must be carefully evaluated. For example, residual PEG from precipitation procedures can produce interference peaks with a 44 Da spacing, severely affecting liquid chromatography with tandem mass spectrometry (LC-MS/MS) analysis [112], while PEG and other surfactants often cause ion suppression [113]. High-salt buffer systems can also reduce detection sensitivity by inhibiting ionization efficiency [114].

## 6 Conclusion and future perspectives

A broad range of classical and emerging EV isolation strategies have been developed for blood-derived samples, including differential UC, SEC, UF, PEG, immunoaffinity capture, AF4, and microfluidic platforms. Each of these methods presents unique advantages and is suited to specific research goals. However, in practice, no single method offers an all-purpose solution: it is difficult for any individual approach to simultaneously optimize purity, yield, specificity, and throughput. As a result, researchers must make trade-offs. Moreover, a lack of comprehensive standardization persists across the EV isolation workflows. Variability in sample preprocessing, operational parameters, and instrumentation contributes to poor inter-laboratory reproducibility and challenges in data comparability. The commonly used techniques still face several limitations, including contamination from non-EV particles, low recovery efficiency, limited scalability, and insufficient capacity for isolating functionally distinct EV subpopulations.

Building on these challenges, the future development of blood-derived EV isolation technologies should aim at meeting diverse research objectives while addressing current limitations in purity, yield, standardization, and scalability. Combined or modular workflows show promise in improving sample quality but require careful balancing of efficiency and quality standards. The next priority will be to foster the development of automated, integrated, and clinically adaptable platforms, particularly those capable of processing low-volume samples and interfacing seamlessly with downstream multi-omics analyses such as MS, RNA-seq, and digital PCR. The ideal approach should be operationally simple, deliver consistent output, demonstrate reproducibility across laboratories, and align with standardized frameworks, thereby accelerating the translation of EVs from basic research to clinical applications and enabling their full potential in early diagnosis, disease stratification, prognosis monitoring, and personalized medicine.

## CRediT authorship contribution statement

**Xi Song:** Writing – original draft, Investigation. **Jacques Crommen:** Writing – review & editing. **Marianne Fillet:** Writing – review & editing, Validation, Supervision, Funding acquisition.

## Declaration of competing interest

The authors declare that they have no known competing financial interests or personal relationships that could have appeared to influence the work reported in this paper.
